# A commentary on ‘Patterns and prognostic values of programmed cell death-ligand 1 expression and CD8+ T-cell infiltration in small cell carcinoma of the esophagus: a retrospective analysis of 34 years of national cancer center data in China’

**DOI:** 10.1097/JS9.0000000000002927

**Published:** 2025-07-07

**Authors:** Yunliang Yu, Ting Li, Ni Li

**Affiliations:** a Department of Medical Laboratory, Yantai Affiliated Hospital of Binzhou Medical University, Yantai, Shandong, China; b Department of Medical Laboratory, Yantai-Mountain Hospital of Yantai, Yantai, Shandong, China

*Dear Editor*,

We read with great interest the recent article titled “Patterns and prognostic values of programmed cell death-ligand 1 expression and CD8+ T-cell infiltration in small cell carcinoma of the esophagus: a retrospective analysis of 34 years of National Cancer Center data in China”^[1]^ published in the International Journal of Surgery. The goal of this study is to investigate the expression profile and clinical implications of programmed cell death-ligand 1 (PD-L1) and CD8+ T cells in SCCE for the first time. The findings indicate that PD-L1 expression was only found in TIICs from roughly 50% of SCCE patients. PD-L1 and CD8 status are new prognostic indicators in SCCEs that could guide the use of risk-related treatment approaches. PD-L1 expression was also increased in SCCEs with stronger CD8 infiltration, indicating the emergence of adaptive immune resistance. Future studies must, however, address a number of important concerns.

First, sample selection limitations. This study is a retrospective analysis that included 147 patients with surgically resected small cell esophageal carcinoma (SCCE) from 1985 to 2019, spanning 34 years. This design may introduce selection bias, particularly since the criteria for sample selection were not clearly specified over time (such as surgical techniques and pathological diagnostic standards). Additionally, the study did not assess the immune characteristics of patients who did not undergo surgery, which could potentially overestimate the prognostic value of PD-L1 and CD8+ T cells. Although the authors noted that patients who received chemotherapy or radiotherapy before surgery were excluded, they did not provide detailed explanations on how other potential confounding factors, such as differences in postoperative adjuvant therapy, might have influenced the results.

Second, PD-L1 detection methods’ limitations^[2,3]^. The study used the E1L3N antibody to detect PD-L1, but its consistency with commonly used clinical antibodies (such as 22C3) has not been fully validated. The authors noted that 22C3 is almost undetectable in samples stored for over 5 years, but they did not provide specific data to support the stability of E1L3N in long-term storage samples. Additionally, the threshold for PD-L1 positivity (TIICs ≥1%, CPS ≥1) lacks clinical validation in SCCE, which may affect the comparability of the results. The study did not explore the potential impact of different antibodies or thresholds on prognosis analysis, limiting the generalizability of its conclusions. Future studies should conduct prospective research to validate PD-L1 detection standards.

Third, statistical analysis and results reporting flaws. In the multivariate Cox regression, the linear assumptions or nonlinear relationships of continuous variables (such as CD8 density) were not addressed. The Kaplan-Meier curve did not present a risk table, and the analysis of interactions between PD-L1 and CD8 was not corrected for multiple testing. Key data, such as baseline characteristics of each subgroup, were not provided in the supplementary materials, which limits the reproducibility of the results. Additionally, the sample size (*n* = 147) was not discussed in terms of its impact on detecting weak associations, such as the HR for PD-L1 and RFS being 0.67, *P* = 0.067, potentially overlooking potentially significant results.

In summary, the study represents an important contribution to the understanding of PD-L1 expression and CD8 + T-cell infiltration in SCCE. However, several critical points need to be considered for future research endeavors to build upon this foundation. Addressing these limitations could refine our understanding of the immune landscape in SCCE and enhance the development of more effective therapeutic strategies. All this was observed regarding the transparency in the reporting of Artificial Intelligence—the TITAN guideline^[4]^.

## Data Availability

No data was used in this Letter to the Editor.
